# A case report of late-onset cerebellar ataxia associated with a rare p.R342W *TGM6* (SCA35) mutation

**DOI:** 10.1186/s12883-020-01964-1

**Published:** 2020-11-07

**Authors:** Arianna Manini, Tommaso Bocci, Alice Migazzi, Edoardo Monfrini, Dario Ronchi, Giulia Franco, Anna De Rosa, Ferdinando Sartucci, Alberto Priori, Stefania Corti, Giacomo Pietro Comi, Nereo Bresolin, Manuela Basso, Alessio Di Fonzo

**Affiliations:** 1 Neurology Unit, Foundation IRCCS Ca’ Granda Ospedale Maggiore Policlinico, Dino Ferrari Center, Neuroscience Section, Department of Pathophysiology and Transplantation (DEPT), University of Milan, Via Francesco Sforza 35, 20122 Milan, Italy; 2 ”Luigi Sacco” Department of Biomedical and Clinical Sciences, University of Milan, Milan, Italy; 3 ”Aldo Ravelli” Center for Neurotechnology and Experimental Brain Therapeutics, Department of Health Sciences, University of Milan and ASST Santi Paolo e Carlo, Milan, Italy; 4 Department of Cellular, Computational and Integrative Biology - CIBIO, University of Trento, Trento, Italy; 5 Department of Clinical and Experimental Medicine, Unit of Neurology, Pisa University Medical School, Pisa, Italy

**Keywords:** Spinocerebellar ataxias, SCA35, *TGM6*, Transglutaminase, Case report

## Abstract

**Background:**

Mutations in *TGM6* gene, encoding for transglutaminase 6 (TG6), have been implicated in the pathogenesis of spinocerebellar ataxia type 35 (SCA35), a rare autosomal dominant disease marked by cerebellar degeneration and characterized by postural instability, incoordination of gait, features of cerebellar dysfunction and pyramidal signs.

**Case presentation:**

Here we report the case of an Italian patient with late-onset, slowly progressive cerebellar features, including gait ataxia, scanning speech and ocular dysmetria and pyramidal tract signs. Whole exome sequencing revealed the rare heterozygous c.1024C > T (p.R342W) variant of *TGM6*, located at a highly evolutionary conserved position and predicted as pathogenic by in silico tools. Expression of TG6-R342W mutant in HEK293T cells led to a significant reduction of transamidase activity compared to wild-type TG6.

**Conclusion:**

This finding extends SCA35 genetic landscape, highlighting the importance of *TGM6* screening in undiagnosed late-onset and slowly progressive cerebellar ataxias.

**Supplementary Information:**

The online version contains supplementary material available at 10.1186/s12883-020-01964-1.

## Background

Spinocerebellar ataxias (SCAs) embody a clinically and genetically heterogeneous group of disorders, characterized by cerebellar degeneration. A broad range of signs and symptoms, from retinopathy to neuropathy, pyramidal signs and epilepsy may be associated with the clinical core picture of cerebellar syndrome. The autosomal dominant inheritance represents a distinctive hallmark. Although pathological repeat expansions are responsible for the majority of presentations (including SCA1, SCA2, SCA3, SCA6, SCA7, SCA8, SCA10, SCA12, SCA17, SCA31, SCA36, SCA37 and DRPLA), an increasing number of SCAs is progressively being associated with conventional mutations (e.g., SCA5 – *SPTBN2*; SCA11 – *TTBK2*; SCA14 – *PRKCG*; SCA28 – *AFG3L2*) [1]. In line with this last group, spinocerebellar ataxia type 35 (SCA35) results from missense mutations in *TGM6,* as found by Wang and colleagues by combining exome sequencing and linkage analysis in four probands of a Chinese family [2]. Since then, several *TGM6* mutations have been described. Some of them, sharing a common reduction in transamidase activity, are thought to be pathogenic, although the specific molecular pattern involved remains uncovered. TG6, a member of the transglutaminase superfamily specifically expressed in the central nervous system, is a calcium-dependent enzyme involved in protein cross-linking. Clinical presentation of SCA35 includes slowly progressive postural instability and incoordination of gait, features of cerebellar dysfunction - hand tremor, dysarthria, dysmetria, and saccadic slowing - and pyramidal signs [3]. Herein, we report the first Italian SCA35 patient with PD family history harbouring a rare *TGM6* variant, predicted as pathogenic by in silico tools and associated with a significant reduction in the transamidase activity in vitro.

## Case presentation

The proband is a 62 year-old-man and has no siblings. His parents were non-consanguineous, both of Italian origin. The father did not suffer from any neurological disorder, while the mother, at the age of 75, received the diagnosis of tremor dominant PD, which was responsive to levodopa. Her main clinical features were rest and postural tremor, involving especially the right upper limb, and hypophonia. She did not show ataxia nor symptoms of autonomic dysfunction during disease, until she died four years later.

No neurological signs or symptoms were reported by the patient until the age of 54, when he began complaining of sialorrhea. Two years later, erectile dysfunction appeared; no other signs or symptoms of dysautonomia, including orthostatic hypotension, urinary incontinence and constipation, appeared in course of disease, thus making unlikely the hypothesis of Multiple System Atrophy type C. Subsequently, a mild dysphagia of solid food was noticed, shortly followed by cerebellar dysarthria and rapidly progressing postural instability, which led to recurrent falls and deambulation loss by the age of 61.

Current clinical examination revealed scanning speech, ocular dysmetria and slow saccades; no nystagmus nor limitation in extraocular movements were observed. Pyramidal tract signs, including limbs hyperreflexia, bilateral extensor plantar responses and ankle clonus, were present. No tremor nor bradykinesia were detected. The patient showed unsteadiness in standing position. He was able to walk without support only for a few steps, revealing spastic-ataxic gait, with tendency to fall backward. The remaining neurological examination, including trophism, strength and sensory testing, were normal.

Severity of cerebellar ataxia was evaluated by using the Scale for the Assessment and Rating of Ataxia (SARA) and the International Cooperative Ataxia Rating Scale (ICARS) at 59 (respectively 18/40 and 44/100) and 61 (respectively 25/40 and 61/100) years old.

**Fig. 1:**
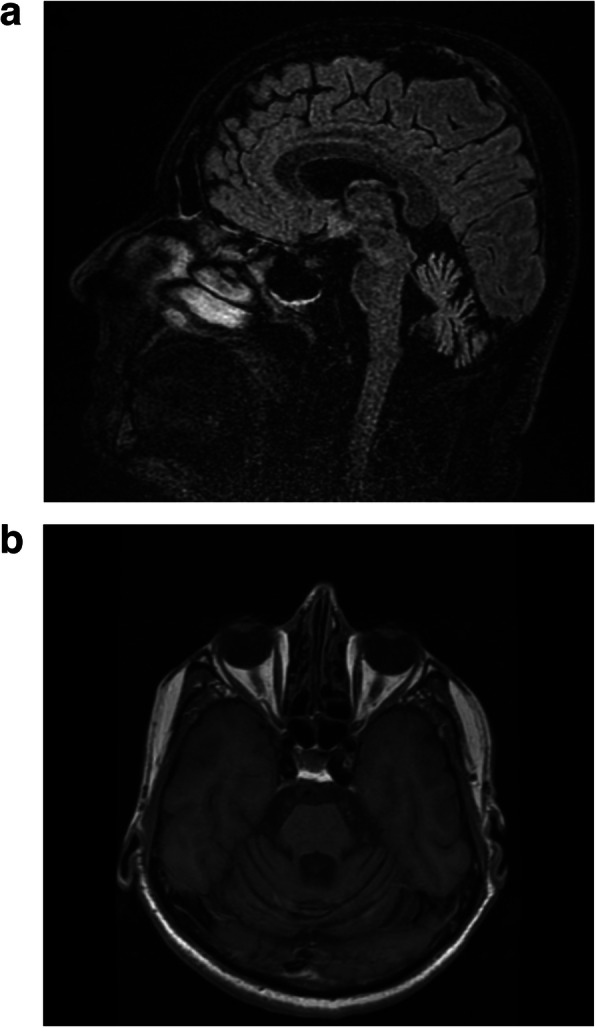
Patient brain MRI. Patient brain MRI performed at the age of 58 years, showing cerebellar atrophy and mild brainstem atrophy. **a**) Sagittal FLAIR image. **b**) Axial T1-weighted image

Cerebellar atrophy and mild brainstem atrophy were detected at brain Magnetic Resonance Imaging (MRI) (Fig. 1a-b). 2-[18F]fluoro-2-deoxy-D-glucose (2-[18F]FDG) Positron Emission Tomography (PET) imaging displayed diffuse hypometabolism of the left cerebellar hemisphere. Nerve conduction studies were normal, while needle examination showed mild signs of bilateral chronic neurogenic damage at the level of tibialis anterior muscles. Tests of cardiovascular and sudomotor function, including heart-rate variability and sympathetic skin responses (SRR) (Additional file 1), excluded autonomic dysfunction. No autoantibodies associated with paraneoplastic neurologic syndromes (antibodies anti-Hu, Yo, Ri, amphiphysin, Ma2-Ta, CV2, SOX1, ZIC4, GAD26) were detected by wide-spectrum antibody assay on serum.

**Fig. 2:**
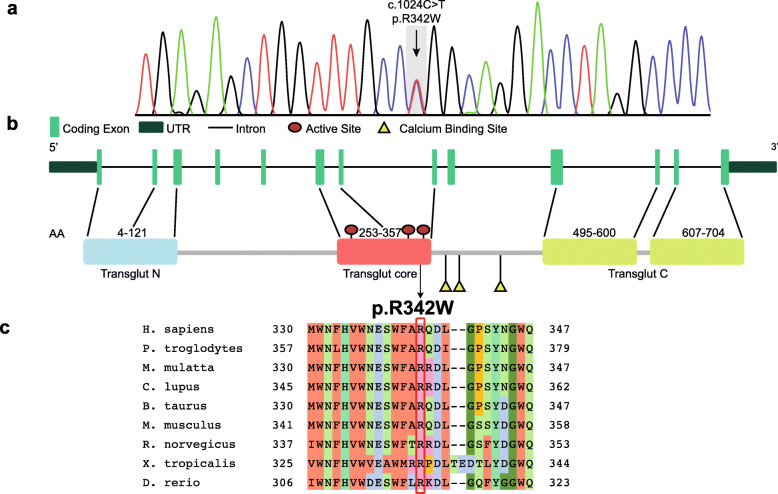
Genetic and bioinformatic analysis. **a** Electropherogram of *TGM6* heterozygous c.1024C > T in the proband. **b** Schematic representation of *TGM6* gene structure and TG6 protein structure, showing the localization of p.R342W. **c** Conservation among orthologous genes of the R342 amino acid (mutation site), with the colours used by the Clustal Omega multiple sequence alignment program (red = hydrophobic, light blue = positively charged, pink = negatively charged, light green = polar, aquamarine = aromatic, dark green = glycine, orange = proline) [4]

After excluding pathological repeat expansions in SCA1–2–3-6-7-8-10-12-17, *ATN1* (DRPLA) and *FXN* (Friedrich Ataxia), Whole Exome Sequencing revealed a heterozygous c.1024C > T, p.R342W mutation in *TGM6* (NM_198994). Nucleotide change was confirmed by Sanger sequencing (Fig. 2a). The identified variant (rs150566697) is rare (gnomAD MAF 0.02%) and replaces a highly conserved arginine in the functional Transglutaminase Core Domain (Fig. 2b-c). The variant is predicted to be damaging by Combined Annotation Dependent Depletion (CADD), Mutation Taster, Sorting Intolerant From Tolerant (SIFT), PolyPhen2, Functional Analysis Through Hidden Markov Models (FATHMM), Mutation Assessor and MutPred2 (Additional file 2). DNA from parents was not available. The variant was not detected in five first degree asymptomatic cousins, respectively of 54, 54, 51, 48 and 46 years old.

**Fig. 3:**
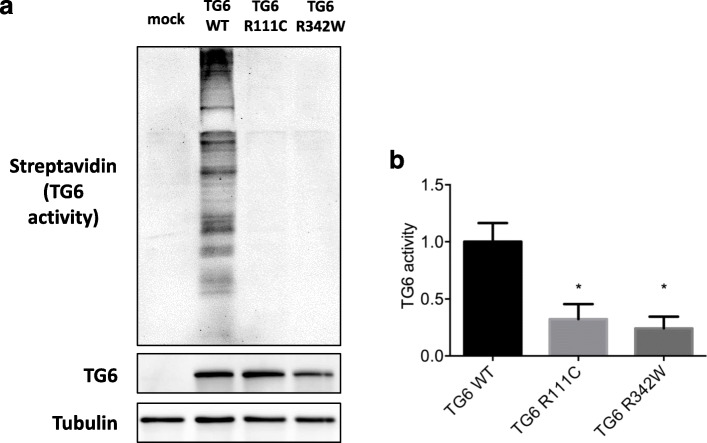
In vitro functional analysis. Significant reduction of transamidase activity of TG6-R342W and TG6-R111C in transiently transfected HEK293T cells, showed by immunoblotting analysis (**a**) and quantification of enzymatic activity (**b**). The transamidase activity was measured by incubating equal amounts of proteins from the cell lysates with a biotinylated peptide (Biotinyl-Thr-Val-Gln-Gln-Glu-Leu-OH 0.5 mM, Zedira GmbH #B001), which is irreversibly cross-linked to proteins in the presence of DTT (5 mM) and high calcium concentration (5 mM CaCl2), and detected by SDS-PAGE electrophoresis followed by western blotting with an anti-streptavidin antibody conjugated to a fluorophore with emission at 800 nm (LI-COR IRDye 800CW #926–32,230, 1:10000). Images were then acquired with an Odyssey infrared imaging system (LI-COR Biosciences). TG6 activity is shown as fold change compared to TG6-WT activity (set as 1). Graph, mean ± SEM, *P < 0.05, 1-way ANOVA with Tukey’s post hoc test. Original uncropped blots are shown in Additional file 3

To investigate the impact of p.R342W on TG6 function, we expressed plasmids encoding the TG6-R342W mutant in HEK293T cells, together with wild-type TG6 (TG6-WT) and TG6-R111C mutant, a known *TGM6* pathogenic variant. TG6-R111C mutant was selected as positive control because it produced a significant lowering of TG6 activity if compared with other variants and altered TG6 subcellular localization, as reported in a previous work [5]. Immunoblotting analysis and quantification of enzymatic activity showed that the transamidase activity of TG6-R342W and TG6-R111C mutants was significantly decreased by 76 and 68% respectively, compared to TG6-WT (Fig. 3a-b; Additional file 3).

## Discussion and conclusion

We described the clinical phenotype of an Italian sporadic cerebellar ataxia patient harbouring a confirmed rare heterozygous missense mutation in *TGM6*. The absence of ataxia in parents suggests the possibility of a de novo occurrence of the mutation, in line with other reports of sporadic SCA35 patients [6], or an autosomal dominant inheritance with incomplete penetrance, which could explain the presence of apparently healthy subjects carrying the p.R342W variant in general population. Furthermore, there is the possibility that these apparently healthy subjects carrying the p.R342W variant might develop the phenotype in the future, since SCA35 is a late-onset disease. A recent study questioned the pathogenicity of *TGM6* mutations in SCA, due to their high prevalence among general population, especially in East Asians. The authors argued that both missense and loss-of-function *TGM6* variants are likely benign, as suggested by their low constraint metrics in gnomAD [7]. Although attention to the risk of genetic misdiagnosis should be paid, lines of evidence supporting the pathogenicity of *TGM6* variants exist. To date, sixteen pathogenic variants have been associated to SCA35 clinical phenotype, including p.R342W (Table 1) [2, 3, 5, 6, 8, 9]. In vitro functional studies failed to reveal a reduction in transamidase activity associated with three of these mutations [5, 8], while four did not underwent in vitro analysis [6, 9, 10]. The remaining nine variants, including p.R342W, share a significant enzymatic activity decrease [2, 3, 5]. In a previous work, TG6-R111C showed an altered subcellular localization and almost complete loss-of-function [5]. Indeed, while TG6 is usually detected in the nucleus and, to a lesser extent, in the perinuclear region, TG6-R111C mostly accumulates in the endoplasmic reticulum (ER). Furthermore, Tripathy and colleagues demonstrated that TG6-R111C increases TG6 degradation via the ubiquitin-proteasome system, induces TG6 insoluble fraction accumulation and reduces the enzymatic activity of TG6-WT when co-expressed in COS-7 cells, thus suggesting a dominant negative loss of function effect [5]. We demonstrated that the effect of TG6-R342W mutant, in terms of enzymatic activity, is similar to TG6-R111C. According to this evidence, it seems reasonable to suppose that p.R342W may act through a similar loss-of-function mechanism. In conclusion, we reported the first Italian case of a patient affected by late-onset cerebellar ataxia and pyramidal tract signs, harbouring a rare *TGM6* variant, affecting an amino acid highly conserved among orthologous genes and predicted as pathogenic by in silico tools. Experiments performed in vitro confirmed a significantly reduced transamidase activity of the TG6 mutant. Despite the low frequency of this form among general population, we suggest considering the screening of *TGM6* in undiagnosed subjects with late-onset cerebellar ataxia and suggestive features.

**Table 1:** List of *TGM6* mutations reported in SCA35 patients

Genomic position	Nucleotide change	Amino acid change	Exon	Type	RefSNP	gnomAD MAF	SCA35 carriers	TG6 activity (functional studies)	Clinical features	Mean age at onset (years)		Study reference
20:2398091	c.1550T>G	p.L517W	10	Missense	rs387907097	0.0001	9	Reduced (in vitro)	Gait ataxia Spasmodic torticollis Cerebellar dysarthria Intentional tremor Dysmetria Pyramidal signs (hyperreflexia, Babinski sign)	44		[2]
20:2381081	c.980A>G	p.D327G	7	Missense	rs387907098	9.6 × 10^−5^	2	Reduced (in vitro)	Gait ataxia Cerebellar dysarthria Intentional tremor Eye movements disturbances (slowness)	41		[2]
20:2398069	c.1528G>C	p.D510H	10	Missense	rs201964784	0.0001	5	Reduced (in vitro)	Gait ataxia Dysmetria Pyramidal signs (hyperreflexia, Babinski sign) Postural and intentional tremor Delayed speech development Mental retardation Cognitive impairment Cerebellar dysarthria Numbness in the extremities Eye movements disturbances (limited extraocular movements, dysmetric saccades) Impaired proprioception	20		[3, 6]
20:2375989	c.331C>T	p.R111C	3	Missense	rs372250159	2.5 × 10^−5^	2	Reduced (in vitro and in vivo)	Gait ataxia Limb ataxia Cerebellar dysarthria Tremor Eye movements disturbances (saccade/pursuit aberrations) Pyramidal signs (hyperreflexia)	23		[3]
20:2411135_2411137	c.1722_1724delAGA	p.E574del	11	Deletion	NA	4.0 × 10^− 6^	1	Reduced (in vitro)	Gait ataxia Cerebellar dysarthria Limb ataxia Tremor Nystagmus Eye movements disturbances (saccade/pursuit aberrations) Pyramidal signs (hyperreflexia)	56		[3]
20:2377270	c.543G>T	p.Q181H	4	Missense	NA	NA	1	Reduced (in vitro)	Gait ataxia Myoclonus Epilepsy	19		[8]
20:2384304	c.1171G>A	p.V391M	9	Missense	rs116904482	0.0008	1	Reduced (in vitro)	Gait ataxia Extrapyramidal signs Dystonia	36		[8]
20:2384455	c.1322A>G	p.Y441C	9	Missense	rs138950659	1.2 × 10^−5^	1	Reduced (in vitro)	Gait ataxia Pyramidal signs (spasticity) Cerebellar dysarthria Dysphagia	54		[8]
20:2397883	c.1342C>T	p.R448W	9	Missense	rs147979536	0.015	1	Not reduced (in vitro)	Gait ataxia	20		[8]
20:2398046	c.1505T>A	p.L502Q	10	Missense	NA	7.1 × 10^−6^	1	Not reduced (in vitro)	Ataxia	> 50		[8]
20:2411658_2411660	c.1951_1952insAAC	p.Q652dup	12	Duplication	NA	0.0013	1	Not reduced (in vitro)	Gait ataxia Myoclonus	NA		[8]
20:2375947	c.288_290delC	p.L97*	3	Frameshift	NA	1.1 × 10^−4^	1	NA	Postural and intentional tremor Cerebellar dysarthria Dystonia Dysmetria Gait ataxia Pyramidal signs (hyperreflexia, Babinski sign)	35		[10]
20:2361622	c.7+1G>T	Splice site change	1	Splicing	NA	1.4 × 10^− 4^	2	NA	Gait ataxia Cerebellar dysarthria Tremor	57		[5]
20:2398019	c.1478C>T	p.P493L	10	Missense	NA	8.0 × 10^− 5^	1	NA	Gait ataxia Cerebellar dysarthria Tremor Cognitive impairment	60		[5]
20:2380376	c.841delC	p.L281*	6	Frameshift	NA	NA	1	NA	Gait ataxia Cerebellar dysarthria Extrapyramidal signs Dysmetria Pyramidal signs (hyperreflexia)	40		[9]
20:2384077	c.1024C>T	p.R342W	8	Missense	rs150566697	0.0002	1	Reduced (in vitro)	Gait ataxia Cerebellar dysarthria Eye movements disturbances (ocular dysmetria, slow saccades) Pyramidal signs (hyperreflexia, Babinski sign, ankle clonus)	54		*Current study*

*truncated protein

## Supplementary Information

**Additional file 1:** Sympathetic Skin Responses (SSR). SSR were simultaneously recorded both from hands and feet, following electrical stimulation delivered over the median nerve at the wrist: stimulation intensity was set at 30 mA for 0.2 milliseconds and three stimuli were delivered at random intervals of more than 1 min to avoid habituation, in accordance with previously described methods [11]. Note that onset and peak-latencies were within normal limits (O: onset-latency; P: peak-latency).

**Additional file 2:** In silico pathogenicity prediction. Assessment of the deleterious impact of the *TGM6* p.R342W variant by the in silico prediction tools CADD, Mutation Taster, SIFT, PolyPhen2, FATHMM, Mutation Assessor and MutPred2.

**Additional file 3:** Original full-length blots for Fig. 3. Original uncropped blots showing the three independent experiments performed to analyse the transamidase activity of TG6-R342W compared to wild-type TG6 (TG6-WT) and TG6-R111C. Red arrow indicates overexpressed TG6. Replicate number 1 was chosen as representative blot for Fig. 3. Each single blot (labelled from A to F) was added on separate pages below.

## Data Availability

Data sharing not applicable to this article as no datasets were generated or analyzed during the current study.

## Abbreviations

SCAs: Spinocerebellar ataxias
NGS: Next generation sequencing
PD: Parkinson’s disease
MRI: Magnetic resonance imaging
2-[18F]FDG: 2-[18F]fluoro-2-deoxy-D-glucose
PET: Positron emission tomography
SRR: Sympathetic skin responses
WT: Wild-type

## References

[1] Durr A (2010). Autosomal dominant cerebellar ataxias: polyglutamine expansions and beyond. Lancet Neurol.

[2] Wang JL, Yang X, Xia K, Hu ZM, Weng L, Jin X, et al. TGM6 identified as a novel causative gene of spinocerebellar ataxias using exome sequencing. Brain 2010;133(12):3510–3518. Available from: https://academic.oup.com/brain/article-lookup/doi/10.1093/brain/awq323. 10.1093/brain/awq323 21106500

[3] Guo Y-C, Lin J-J, Liao Y-C, Tsai P-C, Lee Y-C, Soong B-W. Spinocerebellar ataxia 35: novel mutations in TGM6 with clinical and genetic characterization. Neurol Int 2014;83(17):1554–1561. Available from: http://www.neurology.org/cgi/doi/10.1212/WNL.0000000000000909. 10.1212/WNL.0000000000000909 25253745

[4] McWilliam H, Li W, Uludag M, Squizzato S, Park YM, Buso N (2013). Analysis tool web services from the EMBL-EBI. Nucleic Acids Res.

[5] Tripathy D, Vignoli B, Ramesh N, Polanco MJ, Coutelier M, Stephen CD (2017). Mutations in TGM6 induce the unfolded protein response in SCA35. Hum Mol Genet.

[6] Yang Z, Shi M, Liu Y, Wang Y, Luo H, Wang Z (2018). TGM6 gene mutations in undiagnosed cerebellar ataxia patients. Parkinsonism Relat Disord.

[7] Fung JLF, Tsang MHY, Leung GKC, Yeung KS, Mak CCY, Fung CW (2019). A significant inflation in TGM6 genetic risk casts doubt in its causation in spinocerebellar ataxia type 35. Parkinsonism Relat Disord.

[8] Li M, Pang S, Song Y, Kung M, Ho S-L, Sham P-C. Whole exome sequencing identifies a novel mutation in the transglutaminase 6 gene for spinocerebellar ataxia in a Chinese family. Clin Genet 2013;83(3):269–273. Available from: http://doi.wiley.com/10.1111/j.1399-0004.2012.01895.x. 10.1111/j.1399-0004.2012.01895.x 22554020

[9] Lin C-C, Gan S-R, Gupta D, Alaedini A, Green PH, Kuo S-H. Hispanic Spinocerebellar Ataxia type 35 (SCA35) with a novel Frameshift mutation. Cerebellum 2019;18(2):291–294. Available from: http://link.springer.com/10.1007/s12311-018-0978-6. 10.1007/s12311-018-0978-6 PMC6544358 30229425

[10] Fasano A, Hodaie M, Munhoz RP, Rohani M (2017). SCA 35 presenting as isolated treatment-resistant dystonic hand tremor. Parkinsonism Relat Disord.

[11] De Marinis M, Stocchi F, Gregori B, Accornero N. Sympathetic skin response and cardiovascular autonomic function tests in Parkinson’s disease and multiple system atrophy with autonomic failure. Mov Disord 2000 Nov;15(6):1215–1220. Available from: http://doi.wiley.com/10.1002/1531-8257%28200011%2915%3A6%3C1215%3A%3AAID-MDS1023%3E3.0.CO%3B2-J. 10.1002/1531-8257(200011)15:6<1215::aid-mds1023>3.0.co;2-j 11104208

