# Supplementary figures and images for: A case report of late-onset cerebellar ataxia associated with a rare p.R342W TGM6 (SCA35) mutation
Source: BMC Neurol. 2020 Nov 7;20:408. doi: 10.1186/s12883-020-01964-1 (PMC7648302; doi:10.1186/s12883-020-01964-1)

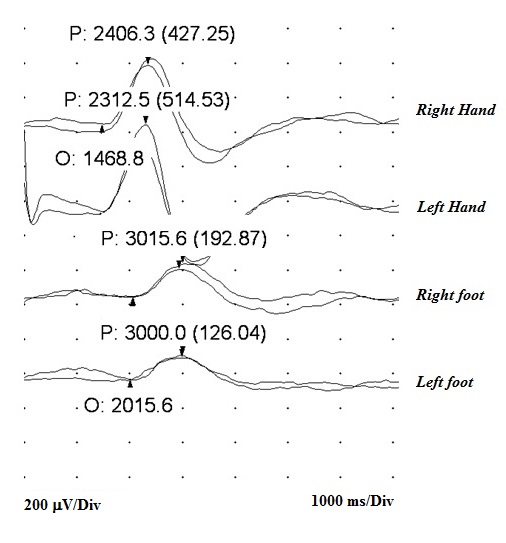

Supplement: Supplementary file 1 — Additional file 1: Sympathetic Skin Responses (SSR). SSR were simultaneously recorded both from hands and feet, following electrical stimulation delivered over the median nerve at the wrist: stimulation intensity was set at 30 mA for 0.2 milliseconds and three stimuli were delivered at random intervals of more than 1 min to avoid habituation, in accordance with previously described methods [11]. Note that onset and peak-latencies were within normal limits (O: onset-latency; P: peak-latency). [file 12883_2020_1964_MOESM1_ESM.jpg]

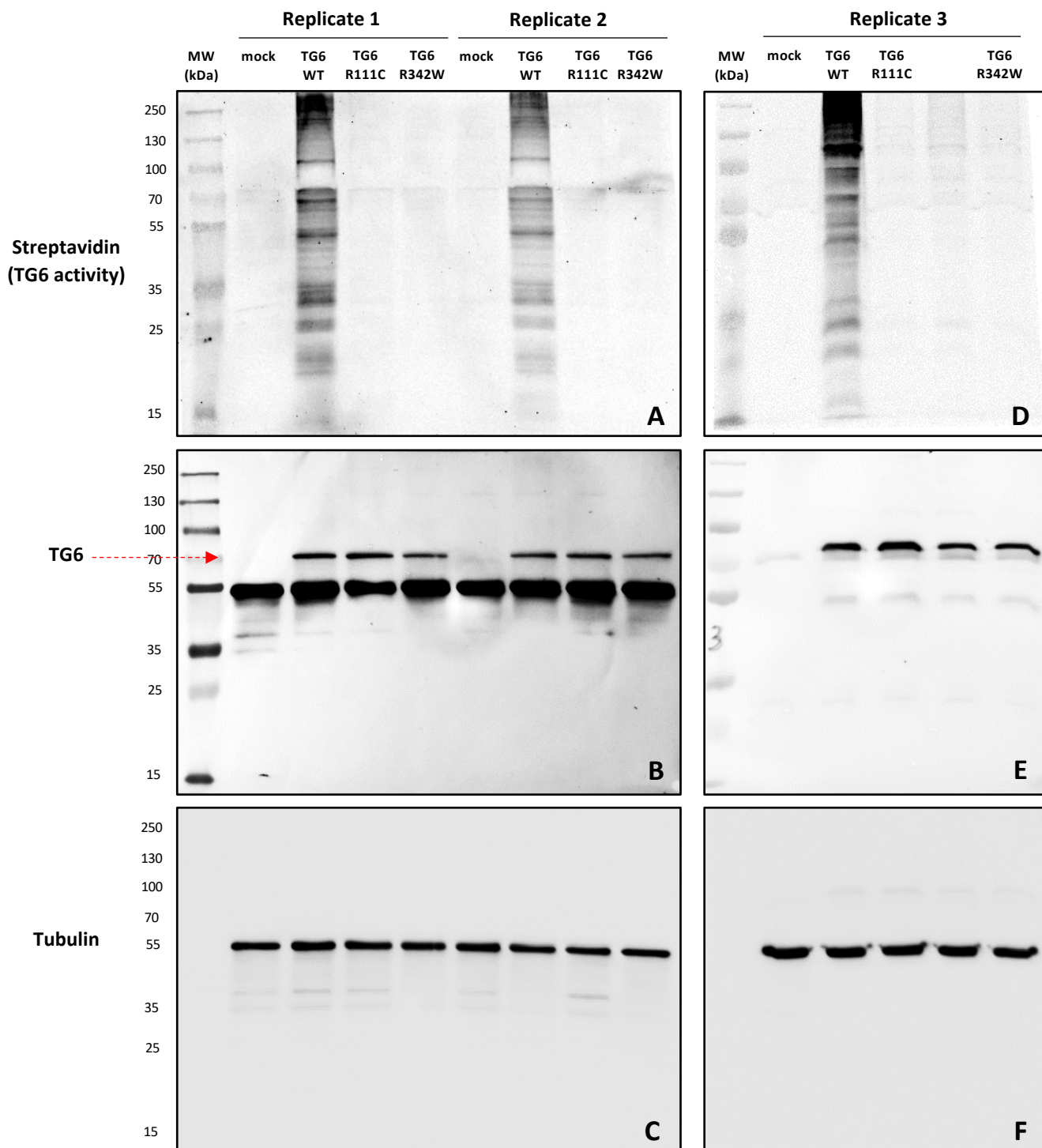

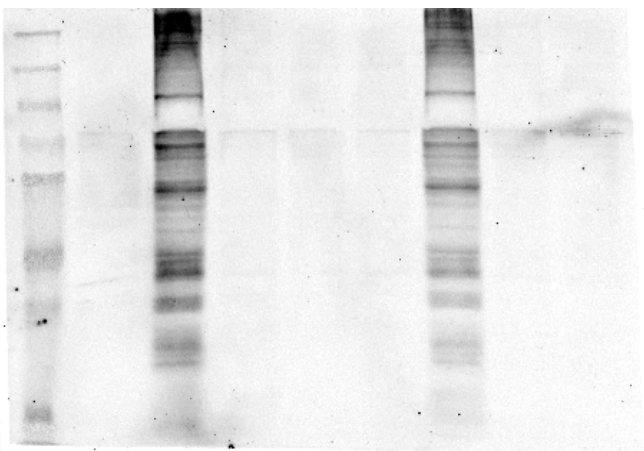

**Blot A**

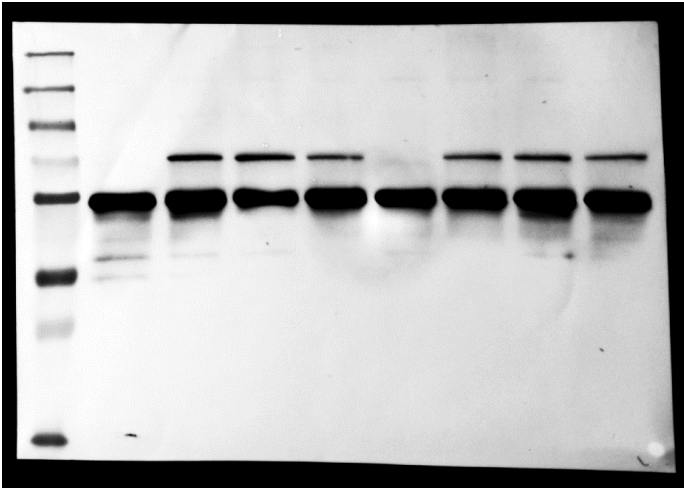

**Blot B**

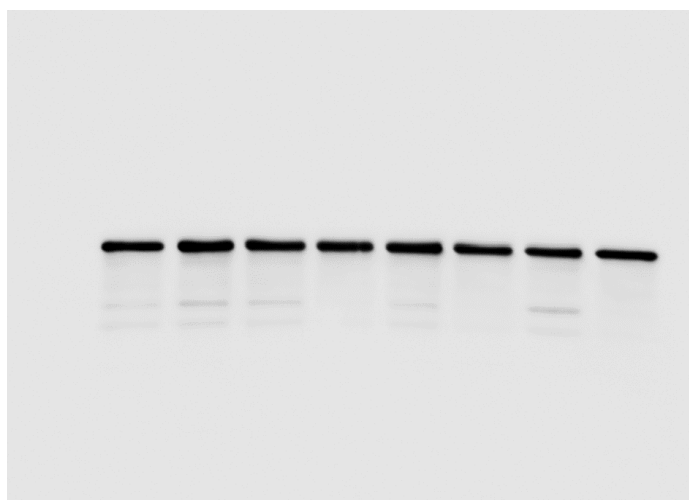

**Blot C**

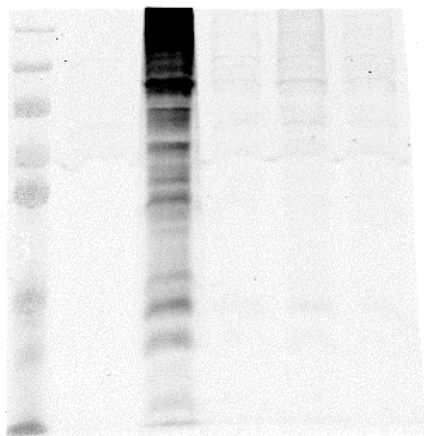

**Blot D**

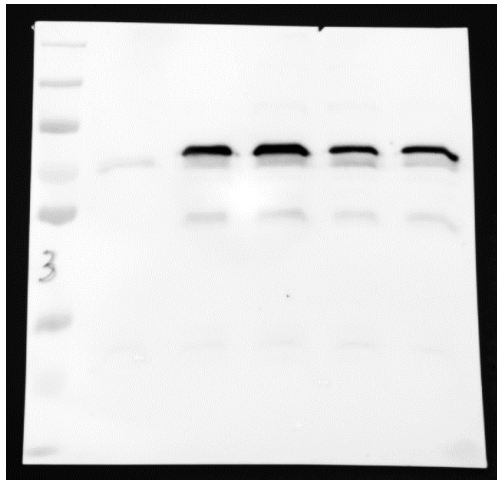

**Blot E**

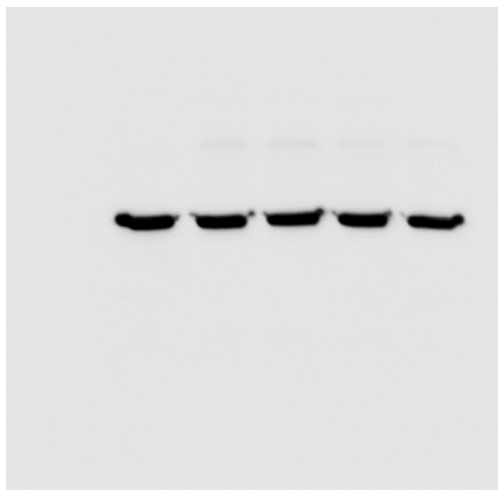

**Blot F**

Supplement: Supplementary file 3 — Additional file 3: Original full-length blots for Fig. 3. Original uncropped blots showing the three independent experiments performed to analyse the transamidase activity of TG6-R342W compared to wild-type TG6 (TG6-WT) and TG6-R111C. Red arrow indicates overexpressed TG6. Replicate number 1 was chosen as representative blot for Fig. 3. Each single blot (labelled from A to F) was added on separate pages below. [file 12883_2020_1964_MOESM3_ESM.pdf]
