# Supplementary material for: A case report of late-onset cerebellar ataxia associated with a rare p.R342W TGM6 (SCA35) mutation
Source: BMC Neurol. 2020 Nov 7;20:408. doi: 10.1186/s12883-020-01964-1 (PMC7648302; doi:10.1186/s12883-020-01964-1)
Supplement: Supplementary file 2 — Additional file 2: In silico pathogenicity prediction. Assessment of the deleterious impact of the TGM6 p.R342W variant by the in silico prediction tools CADD, Mutation Taster, SIFT, PolyPhen2, FATHMM, Mutation Assessor and MutPred2. [file 12883_2020_1964_MOESM2_ESM.docx]

| *TGM6* – c.1024C>T – p.R342W | |
| --- | --- |
| **CADD** | Deleterious  (31) |
| **Mutation Taster** | Disease causing  (0.997) |
| **SIFT** | Damaging  (0.000) |
| **PolyPhen2** | Probably damaging  (1.000) |
| **FATHMM** | Damaging  (-4.01) |
| **Mutation Assessor** | High functional impact  (4.115) |
| **MutPred2** | Pathogenic  (0.880) |
